# AutoStitcher: An Automated Program for Efficient and Robust Reconstruction of Digitized Whole Histological Sections from Tissue Fragments

**DOI:** 10.1038/srep29906

**Published:** 2016-07-26

**Authors:** Gregory Penzias, Andrew Janowczyk, Asha Singanamalli, Mirabela Rusu, Natalie Shih, Michael Feldman, Phillip D. Stricker, Warick Delprado, Sarita Tiwari, Maret Böhm, Anne-Maree Haynes, Lee Ponsky, Satish Viswanath, Anant Madabhushi

**Affiliations:** 1 Case Western Reserve University, Department of Biomedical Engineering, Cleveland OH, 44106, USA; 2 University of Pennsylvania, Department of Pathology, Philadelphia PA, 19104, USA; 3 St. Vincent’s Prostate Cancer Clinic, Darlinghurst, NSW, Australia; 4 Douglass Hanly Moir Pathology, Macquarie Park, NSW, Australia; 5 Garvan Institute of Medical Research/The Kinghorn Cancer Centre, Darlinghurst, NSW, Australia; 6 University Hospitals Seidman Cancer Center, Cleveland OH, 44106, USA

## Abstract

In applications involving large tissue specimens that have been sectioned into smaller tissue fragments, manual reconstruction of a “pseudo whole-mount” histological section (PWMHS) can facilitate (a) pathological disease annotation, and (b) image registration and correlation with radiological images. We have previously presented a program called HistoStitcher, which allows for more efficient manual reconstruction than general purpose image editing tools (such as Photoshop). However HistoStitcher is still manual and hence can be laborious and subjective, especially when doing large cohort studies. In this work we present AutoStitcher, a novel automated algorithm for reconstructing PWMHSs from digitized tissue fragments. AutoStitcher reconstructs (“stitches”) a PWMHS from a set of 4 fragments by optimizing a novel cost function that is domain-inspired to ensure (i) alignment of similar tissue regions, and (ii) contiguity of the prostate boundary. The algorithm achieves computational efficiency by performing reconstruction in a multi-resolution hierarchy. Automated PWMHS reconstruction results (via AutoStitcher) were quantitatively and qualitatively compared to manual reconstructions obtained via HistoStitcher for 113 prostate pathology sections. Distances between corresponding fiducials placed on each of the automated and manual reconstruction results were between 2.7%–3.2%, reflecting their excellent visual similarity.

Whole-mount histological sections (WMHSs) allow for visual and spatial co-registration with pre-operative *in vivo* imaging. This approach can thus allow for spatially mapping disease extent annotated on the *ex vivo* pathology images onto the *in vivo* imaging^1^. However, preparation of whole-mounts is not always feasible in routine clinical practice, as large histological specimens can be difficult to slice thin enough to obtain sections without compromising tissue integrity. In addition to requiring significant amounts of technical expertise and preparation time as well as specialized equipment, whole-mount specimens can be too large to fit on a standard glass microscopy slide. As a result, several clinical centers have adopted the solution of cutting large specimens into multiple smaller fragments, resulting in their examining or annotating multiple slides per section^2^,^3^.

Unfortunately, utilizing tissue fragments spread across multiple slides presents a significant challenge when the pathologic region-of-interest crosses the boundary of the fragments. An example of this is when *in vivo* radiological imagery is cognitively evaluated against corresponding *ex vivo* pathology, in which case it is considerably more demanding to correlate fragmented pathology with whole section *in vivo* MR images. In addition to the visual differences between the two modalities, having to switch between multiple slides to cognitively combine disjoint visual cues makes it difficult to spatially localize tissue fragments directly on imaging. Similarly, when co-registering *ex vivo* pathology and *in vivo* imaging^4^, whole-mount sections are required on both modalities; which are easier to spatially correlate to one another than tissue fragments. Under these circumstances, reconstructing pseudo whole-mount histological sections (PWMHSs) from component tissue fragments fulfills a clear need in pathology annotation and radiology-pathology correlation workflows.

Chappelow *et al*.^2^ presented a semi-interactive computerized tool called HistoStitcher which enabled PWMHS reconstruction from component digitized histological image fragments^2^,^3^. The tool takes as input several user-selected fiducials along the edges of a pair of fragments. A transform to “stitch” the edges together is then computed based on calculating an affine transform (encoding rotation, translation, and scaling) of the image fragments such that the fiducials are brought into spatial alignment. HistoStitcher was shown to be easier to use and more memory-efficient than photo-editing tools such as Photoshop. However, a significant limitation with HistoStitcher lies in its requirement of manual identification of corresponding fiducial points prior to reconstruction. In addition to being time-consuming, this makes HistoStitcher subject to inter- and intra-user variability. In this paper, we present AutoStitcher, an fully automated algorithm for reconstructing whole from image fragments which is able to overcome most current limitations associated with HistoStitcher.

A clear parallel to solving the problem of PWMHS reconstruction may lie in the variety of automated approaches that have been developed for jigsaw puzzle assembly, panoramic photo stitching, and shredded document reconstruction. However, these approaches cannot be directly translated over to our specific problem domain of histological image reconstruction as they make at least one of the following assumptions about the input images:

- Overlap: Most photo stitching algorithms require using images that depict overlapping regions of a common scene, as shown in Fig. 1a, as they operate by aligning matching key-points within these regions^5^,^6^,^7^. Only Poleg and Peleg^8^ have previously presented an algorithm for stitching non-overlapping images, however, it is specifically designed for rectangular photographic images that have no missing pieces.
- Completeness: Most automatic jigsaw puzzle solving algorithms assume that all available puzzle fragment images together depict the entire jigsaw image, implying that none of the pieces are missing (illustrated in Fig. 1c 9,10). Liu *et al*.^11^ have presented an algorithm for assembling hand-shredded photos that does account for missing shreds, however, it relies on matching the boundary contours of adjacent pieces.
- Interlock: As seen in archetypal jigsaw shapes which fit into one another (illustrated by Fig. 1e); this factor is typically accounted for as contours that can be matched based on their curvature. Most automatic jigsaw puzzle solving algorithms make the assumption that the jigsaw fragments “interlock”^9^,^12^. Shredded document reconstruction algorithms focus on reconstructing documents that have been processed by traditional strip- or cross-cut paper shredders^13^, and thus tend to rely on curve matching to identify corresponding shredded fragments^14^.

As depicted in Fig. 1, all of the above assumptions are violated when reconstructing whole-mount histological images. Typically, histological fragments (a) are non-overlapping, since they are cut from a single object; (b) lack completeness as significant tissue loss can occur during pathological processing; and (c) do not interlock, since uneven warping and tissue loss during processing renders boundary contours too dissimilar to match reliably. Further, tissue processing of whole-mount prostates involves quartering and sectioning of a tissue block. This could potentially lead to variable slice depths and orientations between the tissue fragments that are supposed to lie on the same plane^15^. This further exacerbates the lack of interlock, completeness, and overlap between these tissue fragments.

AutoStitcher utilizes a novel cost function that does not make any of the three limiting assumptions of (a) overlapping, (b) completeness, and (c) interlocking images; as previously presented in image reconstruction literature. Our cost function is inspired by two features humans use to perform manual stitching: (i) alignment of similar tissue regions and (ii) contiguity of the prostate boundary. Tissue region similarity is quantified by grayscale intensity histograms, and contiguity of the prostate boundary is quantified by distance between automatically detected points along the boundary of each fragment. AutoStitcher achieves computational efficiency by working hierarchically, utilizing approximate reconstructions performed at lower resolutions to reduce the amount of computation necessary at higher resolutions.

To evaluate AutoStitcher’s performance, 113 sections were stitched both automatically via AutoStitcher and manually via HistoStitcher; which were then qualitatively and quantitatively compared. Quantitative comparison was facilitated via automatically and manually selected fiducial points, as well as Hausdorff distance between reconstruction contours. To investigate whether AutoStitcher could work on an independent cohort from a different institution, parameters were learned on a sub-cohort, and tested on the remaining sections from both institutions.

The remainder of the paper is organized as follows. In the next section, we describe the methodological details of AutoStitcher. Then, we explain our experimental design to evaluate AutoStitcher on histological data from 2 different institutions. Finally, we present and discuss our experimental results, and end with concluding remarks in the last section.

## Methods

### Ethics Statement

Data analysis was waived review and consent by the IRB board, as all data was being analyzed retrospectively, after de-identification. All experimental protocols were approved under the IRB protocol # 02-13-42C with the University Hospitals of Cleveland Institutional Review Board, and all experiments were carried out in accordance with approved guidelines.

### Notation

Notation employed in this paper has been summarized in Table 1. We denote an image *Q* where each image has dimensions [*X*, *Y*], and each pixel in the image has coordinates (*x*, *y*) = {(*x*, *y*)|*x* ∈ [1, *X*] and *y* ∈ [1, *Y*]}.

### Preprocessing and Initialization

**Figure 2:**
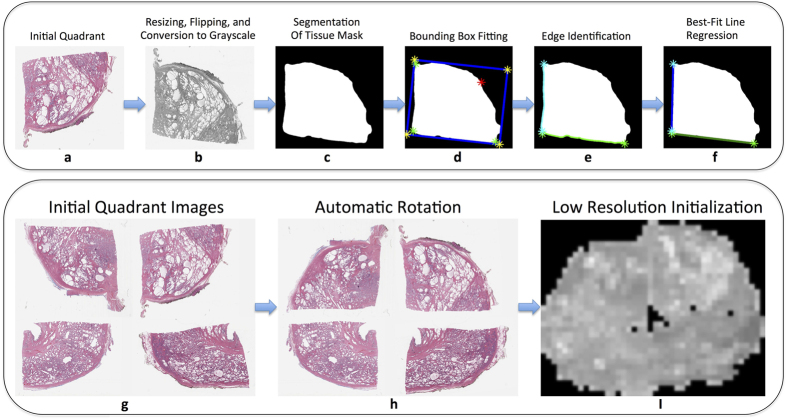
Illustration of AutoStitcher’s preprocessing and low resolution initialization workflow. (**a**) Initial unprocessed quadrant image. (**b**) Resizing, flipping, and conversion to grayscale. (**c**) Segmentation of tissue mask. (**d**) Bounding-box fitting. (**e**) Identification of edges. (**f**) Identification of best-fit lines, followed by automatic rotation. (**g**) Initial unprocessed quadrant images for a single slice. (**h**) Automatically rotated and flipped quadrant images. (**i**) Low resolution initialization.

The only input to AutoStitcher is a set of 4 tissue fragments comprising a single 2D section (selected by a user), which are then pre-processed as follows (see Fig. 2):

- Down-sampling the unprocessed high-resolution tissue fragment images to lower resolution for computational efficiency.
- Applying user-specified flipping to correct for human errors in microscopy slide digitization, where tissue fragments from the same sectioned plane may be scanned on different sides of the original sectioning plane. After flipping, the images are converted to grayscale (depicted in Fig. 2b) to improve computational efficiency. The grayscale pixel values are rescaled from the conventional 8-bit intensity range [0, 255] to decimals within the range [0, 1].
- Segmenting the tissue foreground mask from background, as shown in Fig. 2c, to identify the fragment boundaries.

An approximate initial reconstruction is first computed at low resolution, which is used as an initialization for all subsequent algorithmic processing. Initialization is performed by:

- Computing the minimum-area bounding box, as highlighted in blue in Fig. 2d, and identifying the edges (cyan and green lines in Fig. 2e) as pixels on the tissue boundary contour that fall between “corner” points (green stars in Fig. 2d) closest to the three relevant corners of the bounding box. The fourth corner, highlighted in red, is external to the prostate, so is not used to define an edge.
- Computing best-fit lines to the edges using Theil-Sen linear regression^16^, which achieves robustness to outliers by choosing the median slope of all possible lines through sample points, and rotating the quadrant such that the horizontal best-fit line, as plotted in green in Fig. 2f, is parallel to the x-axis. Figure 2g displays the initially non-rotated quadrants, which is followed by the rotated quadrants in Fig. 2h.
- Finally, the rotated fragments are translated together such that the boundaries of the masks of adjacent quadrants are joined, shown in Fig. 2i.

### Domain-Inspired Cost Function

**Figure 3:**
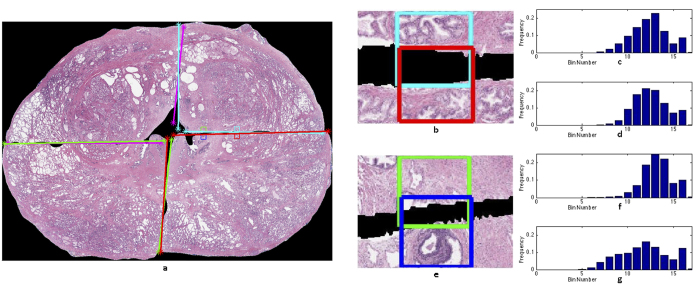
Illustration of the computation of the cost function for AutoStitcher used for stitching at high resolution. (**a**) Stitched image, with two corner points (stars), which are used to compute misalignment *M*, plotted on the ends of each quadrant’s best-fit lines. (**b**) A pair of corresponding patches on quadrants *Q*^*ur*^ and *Q*^*lr*^. (**c**) Intensity histograms of the two patches. Since these patches appear visually similar, their histograms are also similar, thus this pair will have low dissimilarity *D*. (**d**) Another pair of corresponding patches on quadrants *Q*^*ur*^ and *Q*^*lr*^, and (**e**) their intensity histograms, which are more dissimilar, reflecting the apparent visual dissimilarity of their patches.

AutoStitcher uses a two-component, domain-inspired cost function to drive stitching, based on quantifying the dissimilarity of adjacent quadrant regions across the quadrant stitch boundaries, as illustrated in Fig. 3b–g, while the misalignment component quantifies how well the stitched result maintains the continuity of the prostate shape (as illustrated in Fig. 3a).

#### Dissimilarity

Dissimilarity is defined as the degree to which corresponding tissue regions of adjacent quadrants are dissimilar. This is quantified via the following steps:

(i) Identification of corresponding edge-pixels: Corresponding edge pixels are pairs of pixels on adjacent sections that share a common edge, as illustrated by the two pairs of corresponding patches in Fig. 3b,e. They are defined as follows:


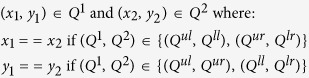


Pixels are defined to be *non-corresponding* if they do not have any corresponding pixel on the adjacent quadrant.

(ii) Patch extraction and histogram (or intensity value) computation: For each corresponding edge-pixel identified in step (i) for any pair of quadrants *Q*_1_ and *Q*_2_, square patches of size *p* are extracted. Intensity histograms with *b* bins are computed from the patches, excluding non-tissue background pixels, which are then normalized by computing the discrete probability density function of the intensity bins. Corresponding histograms (or pixel intensity values) *V*^1^(*k*) and *V*^2^(*k*) are then computed for all *k* ∈ {1, …, *n*}, where *n* is the number of corresponding pixels or patches, such that: *V*^1^(*k*) is the *k*^*th*^ pixel value or histogram vector centered on (*x*_1_, *y*_1_) in *Q*^1^, and *V* ^2^(*k*) is the *k*^*th*^ pixel value or histogram vector centered on (*x*_2_, *y*_2_) in *Q*^2^, such that:


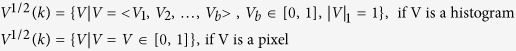


Since the patch size is kept at a constant real-world size, patches encompass only a single pixel at the lowest resolution. Therefore, at the lowest resolution, pixel intensity values are used instead of histograms.

(iii) Dissimilarity computation: While patches of tissue on opposite sides of a cut can appear to be visually different, corresponding patches on either side of the cut can be expected to have more similar intensity distributions compared to image patches that do not correspond. For example, given a cut through a gland-dense region, patches on either side of the cut are likely to have intensity distributions skewed towards higher frequencies of high intensities, since gland lumen are mostly white or very light-colored. This would be reflected in the similarity in their intensity distributions. By contrast, distributions of patches in gland-dense and gland-sparse regions would be very different. Dissimilarity between pairs of histograms (or pixels) is computed using the 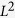-norm of the histogram vector (or intensity value) differences. Given that pixel values are in [0, 1] and histogram vectors are discrete probability density functions that sum to 1, the maximum possible dissimilarity, denoted *ϕ*, between histograms is equal to 2 and between pixels is equal to 1. Non-corresponding pixels are treated consistently by setting their dissimilarity to *ϕ*. The pair-dissimilarity 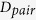 for the pair of adjacent images is thus defined as:


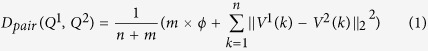


where n is the number of corresponding pixels, and m is the number of non-corresponding pixels.

#### Misalignment

Misalignment is defined as the degree to which adjacent quadrants are incorrectly localized and oriented relative to one another. To compute misalignment, the endpoints of the best-fit lines of the first and second quadrants in the quadrant-pair are identified, as shown in Fig. 3a. The pair of corner points nearest to the outer-boundary of the prostate is denoted 
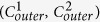
, while the point pair closest to the center of the prostate is denoted 
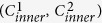
. Misalignment *M*_*pair*_ is computed as:


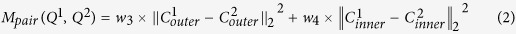


where *w*_3_ and *w*_4_ (*w*_4_ = 1 − *w*_3_) weight the relative contributions of the two pairs of points.

#### Total computation for all pairs of quadrants

AutoStitcher evaluates dissimilarity and misalignment on each of the four pairs of vertically or horizontally adjacent quadrant images (*Q*^*ul*^, *Q*^*ur*^), (*Q*^*ur*^, *Q*^*lr*^), (*Q*^*lr*^, *Q*^*ll*^), (*Q*^*ll*^, *Q*^*ul*^); as follows:


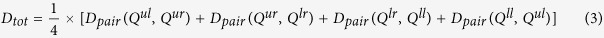



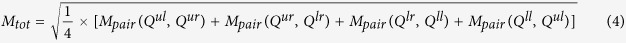


#### Cost Function

The optimal reconstruction is found by determining the set of quadrant transformations **T** of quadrants **Q** that minimizes the cost function below. This cost function involves combining the dissimilarity *D*_*tot*_ and misalignment *M*_*tot*_ in a weighted sum (equation 5), where *w*_2_ = 1 − *w*_1_. The set of quadrant transformations **T** specifies the rigid-body transformations of three moving quadrants relative to the fourth fixed quadrant. Each quadrant’s rigid-body transformation is parameterized by three degrees of freedom (two for translation, one for rotation).


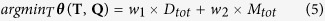


Optimization of equation (5) is done via genetic algorithms for each resolution in the hierarchy. Genetic algorithms were utilized as they are well-suited to such problem domains, that are highly nonlinear and have many local optima^17^. In our implementation, each “generation” of the genetic algorithm comprises a set of 20 solutions, where each solution is a nine-element vector specifying a combination of transformations of three quadrants relative to the fourth quadrant. Optimization concludes after twenty-five consecutive generations that have not demonstrated a significant improvement in the cost function. To ensure computational efficiency, the maximum and minimum possible translations and rotations are restricted to within a local search window of the next lower image resolution.

## Experimental Design

### Data Collection and Processing

Data from prostate cancer patients who underwent radical prostatectomy were acquired from two institutions: (1) 19 patients from University of Pennsylvania, and (2) 17 patients from St. Vincent’s Hospital. Each surgically resected prostate gland was fixed in formalin, embedded in paraffin, and sectioned axially in a plane perpendicular to the urethral axis from apex to base in 3–4 mm sections. Each slice was then sectioned into four quadrants (see Fig. 2g for sample data), stained with hematoxylin & eosin (H & E), and digitized via an Aperio^®^ whole slide scanner at 20X magnification and 0.5/pixel resolution. Digitized slides were de-identified and labeled with the anatomic location of the slide (left- or right-anterior or posterior of the prostate gland).

Our final curated dataset contains a total of 113 sections (452 quadrants) from 36 patients. Note that 143 (out of a total of 256) sections were excluded from our cohort due to (a) large quantities of missing or extra-prostatic tissue (100 out of 143), (b) not having been sectioned into quadrants (e.g., left-to-right-sliced apex or base) (43 out of 143).

All 113 sections were reconstructed into PWMHS via each of AutoStitcher and HistoStitcher. AutoStitcher parameters are described in *parameter selection* in the next subsection. Manual reconstruction via HistoStitcher was performed by a user with 2 years of previous experience in utilizing the software.

### Evaluation

A total of 113 sections were utilized for evaluation, of which 20 were used for parameter selection, and 44 from University of Pennsylvania plus 49 from St. Vincent’s Hospital were used for independent testing.

As there is no “perfect” ground truth whole-mount section for the clinical data utilized in this study, we quantitatively evaluated the accuracy of reconstruction for AutoStitcher with respect to HistoStitcher using three different error measures. Two of the measures are based on fiducial points, which provide an intuitive error measure as well as being computationally efficient for tracking image transformations. Fiducial error is also commonly used in similar applications where automated methods are evaluated with respect to manual gold-standard results, such as medical image registration^18^,^19^,^20^.

**Figure 4:**
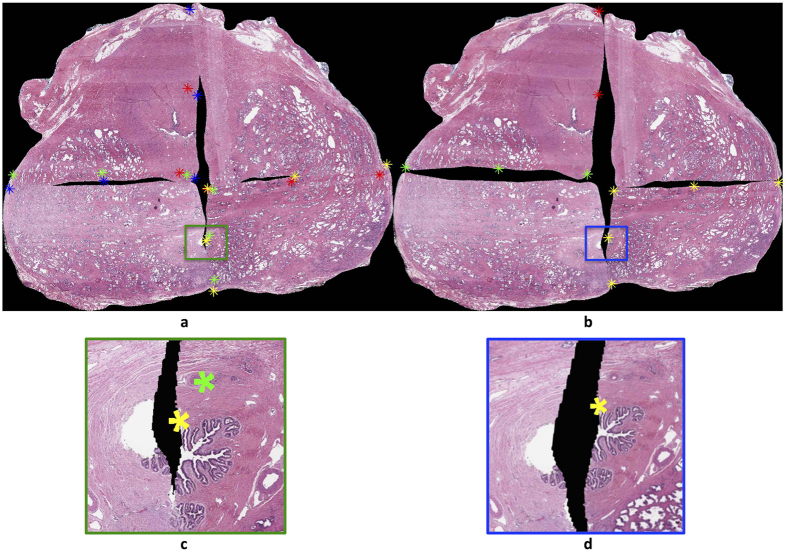
Final reconstruction results of (**a**) AutoStitcher vs. (**b**) HistoStitcher, with ASF evaluation fiducials plotted (via red, green, blue, and yellow asterisks). The enlarged boxed regions in (**c**,**d**) display sample ASF fiducials for each result. Note that since fiducials were selected on the HistoStitcher reconstruction, the panel in (**d**) shows both points being superposed onto the same yellow asterisk, compared to distinct points when mapped onto the AutoStitcher reconstruction in (**c**). Normalized ASF error was computed to be 2.61% in this example.

**Figure 5:**
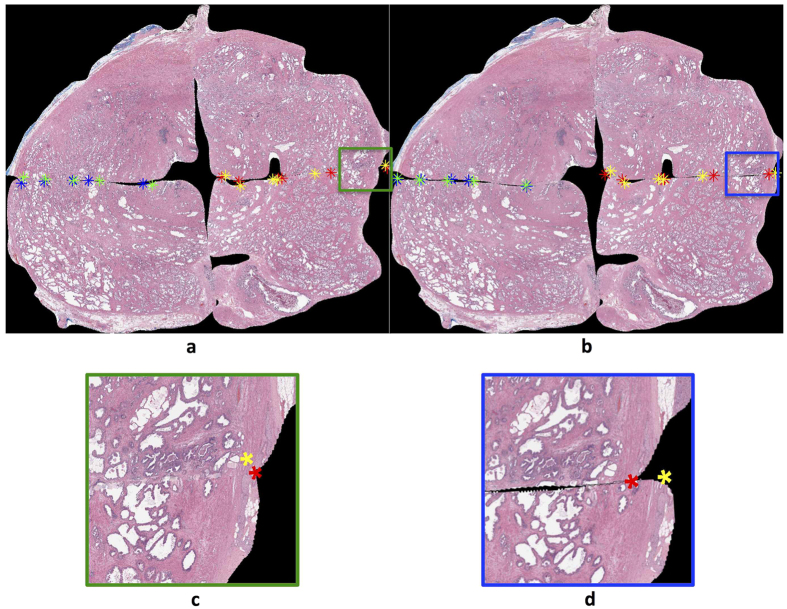
Final reconstruction results of (**a**) AutoStitcher vs. (**b**) HistoStitcher, with MSF evaluation over the fiducials plotted (in red, green, blue, and yellow asterisks). The enlarged boxed regions in (**c**,**d**) display sample MSF fiducials for the AutoStitcher and HistoStitcher reconstructions, respectively. Normalized MSF error was computed to be 1.89% in this example

**Figure 6:**
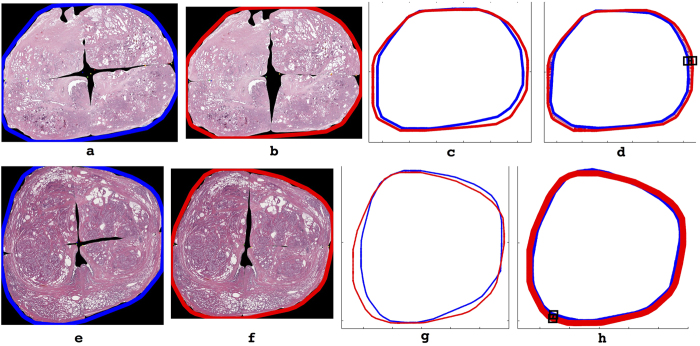
Hausdorff distance evaluation computed on the convex hull of (**a**,**e**) a PWMHS reconstructed using AutoStitcher next to (**b**,**f**) a PWMHS reconstructed using HistoStitcher. The convex hulls are first transformed to a common coordinate system as shown in (**c**,**g**), then aligned such that the Hausdorff distance is minimized in (**d**,**h**). The location of the maximum Hausdorff distance is highlighted in the black boxes, resulting in normalized Hausdorff errors of 3.81% (**d**) and 2.43% (**h**).

Note that in addition to computing these measures in micrometers (μm), we additionally normalized these evaluation measures by the average of length and width of the HistoStitcher reconstruction, to yield a percentage error. In such a case, an error of 100% would result from an AutoStitcher reconstruction containing fiducial points extremely far from where they were positioned on the corresponding HistoStitcher reconstruction. This stitched result would also appear to be overwhelmingly and obviously inaccurate. Similarly, an error of 0% would result from an AutoStitcher reconstruction that is completely identical to the corresponding HistoStitcher reconstruction. It should be noted that an error of 0% does not necessarily represent a perfect result, but simply that the AutoStitcher and HistoStitcher yielded an identical PWMHS reconstruction. Error measures utilized in this study were:

- Automatically selected fiducials (ASF) error: A total of ten pairs of corresponding fiducial points are automatically identified on the endpoints and midpoints of the edges of the *HistoStitcher* reconstruction. Although there are a total of twenty fiducial points (ten pairs), only ten are visible since there is zero distance between each pair as shown in the green, red, and yellow stars in Fig. 4b. These points are then mapped onto the *AutoStitcher* reconstruction, revealing twenty visible points as shown in Fig. 4a. The ASF error is computed for each AutoStitcher reconstruction as the mean distance between all 10 pairs of points (in μm).
- Manually selected fiducials (MSF) error: A total of nine or more pairs of fiducial points were identified by an expert on each HistoStitcher reconstruction. These were selected based on visually identified corresponding landmarks or regions of apparent similarity on the reconstructed PWMHS. Utilizing MSF points thus provides an error measure complementary to the ASF by providing a domain knowledge-based measure, which more accurately reflects an expert’s judgment of the reconstruction quality than the automatically identified ASF points. These points are mapped onto the AutoStitcher reconstruction as depicted in Fig. 5a. The MSF error is computed as the mean distance between all identified point pairs, for each AutoStitcher reconstruction.
- Hausdorff distance (H): A stitched result that maintains the expected outline shape is particularly desirable because this information forms a crucial reference to guide manual selection of anatomic fiducials during image registration. We measure the similarity between shapes of the AutoStitcher reconstruction and HistoStitcher reconstruction outline contours via the Hausdorff distance^21^, The steps involved in the computation are as follows:

1. Binary tissue masks of both the manually and automatically stitched images are obtained from the segmentations performed during pre-processing.
2. The convex hull is computed for each of these masks, as shown in Fig. 6a,b, in order to eliminate concavities in the reconstruction outlines. Note that using the original reconstruction outlines would produce large Hausdorff distances despite visually similar reconstructions.
3. The convex hulls are mapped to a common coordinate system (Fig. 6c).
4. The convex hulls are rotated and translated such that they are maximally aligned, such that the Hausdorff distance between them is minimized (Fig. 6d).
5. The Hausdorff distance is calculated as the distance between points at which the two aligned convex hulls are farthest apart. An ideal Hausdorff distance of zero would occur when the outlines are identical, and thus, the more dissimilar the outlines, the larger the Hausdorff distance.

#### Parameter Selection

Optimal parameter values for AutoStitcher were experimentally determined on a training subset of data consisting of 20 sections from 9 patients from the University of Pennsylvania (see Table 2). Based on the likely ranges for each parameter, optimal parameters were identified as those that produced the minimum error of ASF on the training set, over a total of 72 experiments comprising every possible combination of the parameters *w*_1_, *w*_2_, *w*_3_, *w*_4_, and *p*. Parameter *b* was determined empirically and fixed prior to selection of the other parameters in order to restrict the number of degrees of freedom of the parameter space and make the problem computationally feasible. In synthetic testing (not shown), *b* was not found to significantly affect the reconstruction accuracy. The value for this parameter was picked such that different-looking patches were distinguishable via their histograms while ensuring that the histograms were not sparse.

#### Multi-site evaluation

Learned parameters from the “training” subset of 20 sections were kept fixed when stitching the remaining 93 sections. Inter-institutional variability of AutoStitcher could then be evaluated by comparing ASF error, MSF error, and Hausdorff distance in terms of (a) training error on 20 sections (9 patients) from UPenn, (b) testing error on 44 sections (17 patients) from UPenn, and (c) validation error on 49 sections (17 patients) from St. Vincent’s Hospital. Note that data used to compute testing and validation error was not used in any way to optimize the algorithmic parameters.

Equivalence testing^22^ was utilized to compare each of the three evaluation measures described in the previous subsection over all reconstructions, with the null hypothesis being that evaluation measures were not statistically significantly equivalent between the two institutions. To perform this test, the error distributions were confirmed to be normal using the Kolmogorov-Smirnov test. The mean and standard deviation of the errors were computed, and then a 90% confidence interval relating to the difference between the means of the errors on the testing and validation cohorts was computed. The equivalence margin *δ* was set to 1%, which was estimated to be the smallest difference in mean errors between the two institutions that would render them meaningfully different. If the 90% confidence interval fell within the equivalence margin bounds [−*δ*, *δ*], the error measure was considered to statistically equivalent between the testing and validation cohort at a significance level of *α* = 0.05.

#### Speed Comparison

We estimated the required time of stitching for novice and expert users by surveying three users of HistoStitcher for the typical length of time required to perform manual reconstruction of a PWMHS. Additionally, we measured the length of time required for AutoStitcher to perform automated reconstruction of each PWMHS. Based on these times, we estimated an approximate range of times required for each of (a) the novice users and (b) expert users to perform stitching using (i) AutoStitcher and (ii) HistoStitcher, respectively.

## Results and Discussion

### Experiment 1: Qualitative and Quantitative Evaluation of Reconstruction Accuracy in terms of fiducial error

Figures 4 and 5 illustrate a high degree of apparent visual similarity between the AutoStitcher and HistoStitcher reconstructions (depicted for 2 different sections from 2 different patients). This qualitative similarity is supported by ASF and MSF errors of under 3% (see Table 3), over all 113 sections. In the absence of a true “gold standard” PWMHS reconstruction for the real world clinical data used by us in this study, a fiducial-based error of less than 3% indicates that the AutoStitcher reconstruction is highly similar to the HistoStitcher result even when considering tissue regions that are in correspondence across a stitched edge. It should be noted that our HistoStitcher reconstructions were performed by only a single operator. This was a study limitation and in future work we will look to perform multi-user studies to evaluate the extent of inter-user variability in the use of the AutoStitcher tool.

**Figure 7:**
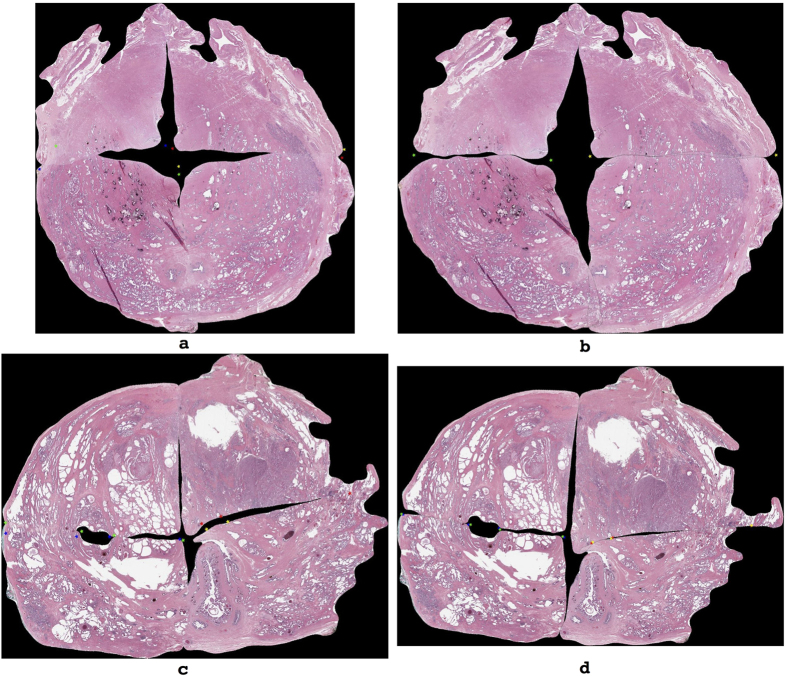
Visual inspection of PWMHSs stitched by (**a**,**c**) AutoStitcher and (**b**,**d**) HistoStitcher, to reveal common causes of reconstruction error: (i) misalignment of quadrants, (ii) under-compensation for missing tissue gaps, (iii) lack of scaling, and (iv) excessive overlap. AutoStitcher reconstruction in panel (**a**) has MSF, ASF, and Hausdorff errors of 6.56%, 5.85%, and 5.46% compared to corresponding HistoStitcher reconstruction in Fig. 7b. AutoStitcher reconstruction in Fig. 7c has MSF, ASF, and Hausdorff errors of 5.51%, 3.27%, and 5.70% compared to HistoStitcher reconstruction in Fig. 7d.

Visual inspection of some of the less similar-appearing reconstructions reveals common patterns among sections where AutoStitcher’s performance deviates from that of HistoStitcher (illustrated in Fig. 7). These include:

- Misalignment, which occurs when regions on adjacent quadrants with similar appearance do not correctly line-up in the reconstructed image. This is depicted in Fig. 7a, in which the lower-left quadrant is misaligned relative to the lower-right and upper-left quadrants. The correctly aligned quadrants are shown in the corresponding HistoStitcher reconstruction in Fig. 7b, based on the operator correctly identifying where the edges should line up between quadrants. Note that misalignment could also be caused by variation in the depth of tissue sectioning and the orientations of quadrants within a section^15^. This is because regions on adjacent quadrants that come from significantly different depths are less likely to have a similar appearance.
- Over-compensation for missing tissue gaps which occurs when there are large portions of tissue missing from the fragments, and AutoStitcher stitches the quadrants together too tightly. In Fig. 7a, the result of over-compensation by the algorithm is apparent in the marked closeness of the lower-left and lower-right quadrants, which contrasts with the significant gap left between these two quadrants in the HistoStitcher reconstruction shown in Fig. 7b. While missing tissue is accounted for in HistoStitcher reconstruction by the operator visualizing how the gaps would appear on a PWMHS, this remains one of the main sources of errors in the case of AutoStitcher.
- Lack of scaling of quadrants by AutoStitcher, as this has not been been implemented in the current version of the algorithm. This is evident in the differences in sizes of the upper-right and lower-right quadrants in the AutoStitcher reconstruction shown in Fig. 7c and the HistoStitcher reconstruction shown in Fig. 7d.
- Excessive overlap of adjacent quadrants, as both HistoStitcher and AutoStitcher allow for some overlap to ensure optimal alignment of quadrants. Despite the fact that there can theoretically be no overlap between quadrant images, both HistoStitcher and AutoStitcher allow for overlap as they utilize only rigid-body transformations when reconstructing a PWMHS. An example of excessive overlap can be seen in the upper-left and lower-left quadrants in the AutoStitcher reconstruction, shown in Fig. 7c. In the HistoStitcher reconstruction shown in Fig. 7d, there is considerably less overlap between these quadrants, based on the operator manually selecting fiducials that would ensure this.

### Experiment 2: Qualitative and Quantitative Evaluation of Reconstruction Accuracy in terms of Hausdorff Distance

Figure 6 illustrates the high degree of visual similarity between the reconstruction outlines of AutoStitcher (blue) compared to HistoStitcher (red). These visualizations are supported by a median normalized Hausdorff Distance of 3%, over all 113 sections (see Table 3), indicating that the reconstruction outlines are highly similar between AutoStitcher and HistoStitcher.

Further inspection of the HistoStitcher reconstructions shown in Fig. 6b, d indicates that they appear slightly wider than the automated reconstructions in Fig. 6a,c. This may be because the HistoStitcher reconstructions allow for more space near the center of the PWMHS to account for missing tissue. As discussed in section the previous subsection and illustrated in Fig. 7, misalignment of tissue fragments and differences in the handling of missing tissue between AutoStitcher and HistoStitcher account for the majority of AutoStitcher’s error.

### Experiment 3: Multi-Site Evaluation

**Figure 8:**
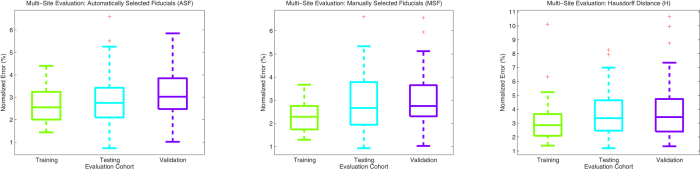
Box and whisker plots of evaluation results between the training cohort, the testing cohort, and the validation cohort, with boxes marking the median, lower-quartile, and upper-quartile, dashed-lines connecting the extremes; and crosses marking outliers. Boxes are colored green, cyan, and purple to distinguish between the three cohorts.

AutoStitcher’s performance was not found to be significantly different between the two institutions for all measures (Table 3). The errors were seen to be marginally higher across all evaluation measures on the validation dataset from St. Vincent’s hospital. As depicted in Fig. 8 via box-and-whisker plots comparing the training, testing, and validation cohorts, ASF and MSF errors have similar ranges between all 3 cohorts. However, the Hausdorff distance errors have noticeably larger ranges and are slightly higher on average on the testing and validation cohorts, compared to the training cohort. This can be explained by the fact that the Hausdorff measure is computed based on a single pair of points, and uses the distance between points at which the aligned outlines are furthest apart, whereas the ASF and MSF are computed based on averages of several pairs of points.

### Experiment 4: Comparison of required time for AutoStitcher vs. HistoStitcher

As shown in Table 4, a novice user of HistoStitcher can take as little as 40 or as long as 80 minutes to complete a stitch (depending on how many times they repeat the process to get a reasonably appearing reconstruction), while an experienced user can perform a stitch in as little 10–20 minutes since they require fewer repetitions. On the other hand, the time required for AutoStitcher to perform a stitch is independent of the level of user experience, and is thus consistently between 4–10 minutes of computation time alone. This is because while AutoStitcher requires only that the user input the image files of each quadrant, and specify whether to flip if quadrants were incorrectly flipped relative to one other during scanning and digitization (which takes up to 30 seconds on average), HistoStitcher requires significantly more user interaction and laborious point selection. When using HistoStitcher, users identify a minimum of nine total pairs of initial corresponding fiducial points to stitch two pairs of quadrants and a pair of hemispheres. After identifying an initial set of corresponding points, they may need to repeat the process by modifying the set of points many times to obtain a satisfactory final reconstruction. Further, this process of point selection has to be repeated for each pair of quadrants and hemispheres.

By comparison, once the set of fragments comprising a single section are identified for AutoStitcher reconstruction, the process of stitching them together is fully automated. Therefore, while a stitching time of 4–10 minutes per section for AutoStitcher vs. 10–20 minutes per section for HistoStitcher may seem like a moderate improvement, the user-interaction time for AutoStitcher is negligible relative to that of HistoStitcher. To provide some context, while a pathologist or researcher seeking to reconstruct a dataset consisting of one-hundred sections using HistoStitcher might spend 2 hours per day at 15 minutes per section and thus take a total of 2.5 weeks to stitch the entire dataset, the same dataset could be automatically reconstructed by AutoStitcher in under 12 hours at 7 minutes per section, with a majority of that time being spent on computation.

## Concluding Remarks

We have presented an automated program called AutoStitcher, which robustly and efficiently digitally stitches pseudo whole mount histological sections from multiple smaller tissue fragments with an accuracy and quality consistent with previous semi-automated methods^2^. Reassembly of whole histological sections from smaller tissue fragments allows for visual and spatial co-registration with pre-operative *in vivo* imaging. This approach can thus allow for spatially mapping disease extent annotated on the *ex vivo* pathology images onto the *in vivo* imaging. AutoStitcher is the first attempt at an algorithm for automated stitching of histology images, and is fundamentally different from algorithms used in similar problem domains in that it makes none of the assumptions of (a) overlap, (b) completeness, and (c) interlock. The algorithm utilizes a novel cost function that performs alignment using a combination of (i) a quantitative measure of image similarity and (ii) automatically detected fiducial points, and achieves computational efficiency on high-resolution histology images by optimizing this function hierarchically at multiple resolution levels. AutoStitcher resulted in PWMHS reconstructions that were evaluated as quantitatively and qualitatively similar to reconstructions performed manually by humans (via HistoStitcher), as measured by human-selected and automatically detected fiducial points as well as the difference between reconstruction contours (difference error of 3% for all measures considered, over 113 PWMHS reconstructions). Our experimental design included separate training and testing cohorts (from 1 institution) and independent validation of the method and parameters on data from a different institution. The differences in the performance of AutoStitcher on data from across the 2 institutions was statistically equivalent to within 1%. AutoStitcher requires approximately 4–10 minutes to reconstruct a single section with a negligible amount of user-interaction, while an expert performing manual stitching using HistoStitcher requires 10–20 minutes of user-interaction. When considering large data cohorts that require reconstruction of large numbers of sections, AutoStitcher could thus save substantial amounts of time and substantially reduce user error.

In future work, the accuracy and efficiency of AutoStitcher could potentially be improved by (a) exploring a larger set of image features for computing region dissimilarity, (b) making the localization of corresponding points across the tissue cut boundary even more robust by incorporating additional domain-information into the cost function, (c) using more sophisticated data-driven statistical methods of incorporating prior knowledge of the prostate capsule and urethra shape, such as active shape models, (d) explicitly detecting areas where tissue is missing, (e) correcting for tissue scaling and nonlinear warping by adding more degrees of freedom to the optimization function, and (f) comparing reconstructions of AutoStitcher to a reference standard with intact tissue (such as high resolution *ex vivo* MR prior to sectioning). These methods could potentially allow sections with large quantities of missing or extra tissue to be stitched more accurately. Although histology sections are likely to undergo scaling and nonlinear warping during tissue processing, in this work we have focused on stitching quadrants together using only rigid transformations. This is because simultaneously solving for just rigid transformations of four individual quadrants was already a highly complex computational problem with nine degrees of freedom. Finally, AutoStitcher could further be validated to work with other types of histological data such as breast or kidney pathology specimens.

## Additional Information

**How to cite this article**: Penzias, G. *et al*. AutoStitcher: An Automated Program for Efficient and Robust Reconstruction of Digitized Whole Histological Sections from Tissue Fragments. *Sci. Rep.* **6**, 29906; doi: 10.1038/srep29906 (2016).

## Figures and Tables

**Figure 1:**
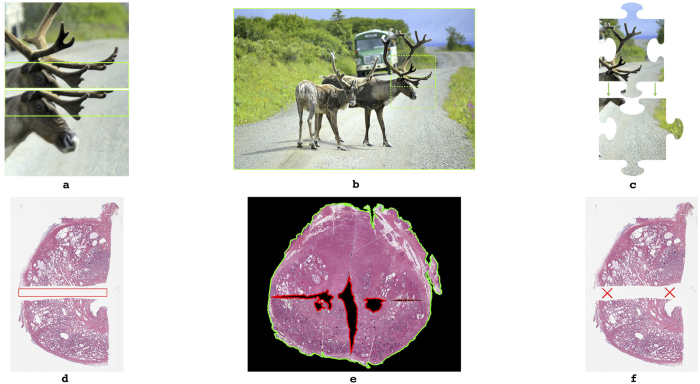
Illustration of assumptions made in related problem domains in image assembly and document reconstruction. (**a**) A pair of overlapping images of a natural scene (green box highlights region of overlap). (**b**) Photograph of a natural scene with no missing pieces (whole image highlighted in green, stitched subimages boxed in dashed lines). (**c**) Interlocking archetypal jigsaw puzzle pieces (green arrows highlight interlocking edges). (**d**) Two corresponding prostate histology quadrants, which do not overlap (red box highlights region of non-overlap). (**e**) A stitched PWMHS which lacks completeness (whole image outlined in green, missing pieces outlined in red). (**f**) Non-interlocking prostate histology quadrants (red X’s highlight lack of interlocking edges).

**Table 1:** Notation.

Symbol	Description
**Q** = {*Q*^*ul*^, *Q*^*ur*^, *Q*^*ll*^, *Q*^*lr*^}	The set of quadrant images corresponding to the upper-left, upper-right, lower-left, and lower-right quadrants, respectively.
**T** = {*T*^*ul*^, *T*^*ur*^, *T*^*ll*^, *T*^*lr*^}	The set of transformations of all 4 quadrant images, where each quadrant’s transformation stores the x-translation, y-translation, and degrees of rotation.
*D*_*pair*_(*Q*^1^, *Q*^2^)	Pair-wise intensity-based dissimilarity computed on each quadrant pair (*Q*^1^, *Q*^2^).
*M*_*pair*_(*Q*^1^, *Q*^2^)	Pair-wise distance-based misalignment computed on each quadrant pair (*Q*^1^, *Q*^2^).
{*V*^1^(*k*), *V*^2^(*k*)}	The *k*^*th*^ set of corresponding histogram vectors or pixel values *V*^1^ and *V*^2^ of a pair of quadrants, where *V*^1^ belongs to *Q*^1^ and *V*^2^ belongs to *Q*^2^.
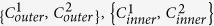	The set of corresponding outer and inner corner points *C*^*1*^ and *C*^*2*^ of a pair of quadrants, where *C*^*1*^ *belongs to Q*^*1*^ *and C*^*2*^ *belongs to Q*^*2*^.
*w*_1_, *w*_2_	Empirically determined cost function component weights (*w*_1_, *w*_2_)
*w*_3_, *w*_4_	Inner- and outer-point weights (*w*_3_, *w*_4_)
*m*, *n*	*m* = Number of overhanging, and *n* = number of corresponding pixels for a pair of adjacent edges
*p*, *b*	*p* = Size of rectangular patch, and *b* = number of histogram bins for cost computation
*ϕ*	Dissimilarity for a non-corresponding edge pixel

**Table 2:** Parameter Selection.

	w_1_	w_2_ = 1 − w_1_	w_3_	w_4_ = 1 − w_3_	p	b
**Optimal Value**	0.989	0.011	0.4	0.6	81 × 81	16
**Range**	0.98–0.995	0.005–0.02	0.3–0.5	0.5–0.7	41 × 41–121 × 121	6–20

**Table 3:** Multi-Site Evaluation.

	University of Pennsylvania (Training)	University of Pennsylvania (Testing)	St. Vincent’s Hospital (Validation)	Cumulative	Inter-Institutional Difference 90% Confidence Interval (University of Pennsylvania (test) vs. St. Vincent’s Hospital).
**Number of Patients**	9	17	17	36	
**Number of Sections**	20	44	49	113	
**Median PWMHS Size** (μm) (Width × Height)	39,382 × 31,216	39,768 × 31,796	41,609 × 34,043	40,750 × 33,080	
**Median Absolute Error (ASF)**	945.45	974.64	1164.4	1061.6	
**Median Normalized Error (ASF)**	2.55%	2.75%	3.03%	2.89%	Statistically equivalent [−0.61%, 0.16%]
**Median Absolute Error (MSF)**	764.41	988.71	1172.3	976.24	
**Median Normalized Error (MSF)**	2.29%	2.67%	2.75%	2.70%	Statistically equivalent [−0.50%, 0.30%]
**Median Absolute Error (H)**	1014.3	1153.7	1348.3	1141.3	
**Median Normalized Error (H)**	2.85%	3.36%	3.44%	3.20%	Statistically equivalent [−0.80%, 0.50%]

**Table 4:** Time to stitch a single section using AutoStitcher vs. HistoStitcher.

	Novice	Expert
**HistoStitcher**	40–80 minutes	10–20 minutes
**AutoStitcher**	4–10 minutes	4–10 minutes
